# Millimeter-Wave Antennas for 5G Wireless Communications: Technologies, Challenges, and Future Trends

**DOI:** 10.3390/s25175424

**Published:** 2025-09-02

**Authors:** Yutao Yang, Minmin Mao, Junran Xu, Huan Liu, Jianhua Wang, Kaixin Song

**Affiliations:** 1 College of Electronic Information and Engineering, Hangzhou Dianzi University, Hangzhou 310018, China; 23040435@hdu.edu.cn (Y.Y.); 23040539@hdu.edu.cn (J.X.); hliu66@hdu.edu.cn (H.L.); kxsong@hdu.edu.cn (K.S.); 2 School of Electronic Engineering, Hangzhou Dianzi University Information Engineering College, Hangzhou 311305, China; wangjianhua@hziee.edu.cn

**Keywords:** 5G communications, millimeter-wave (mmWave) antennas, MIMO, beamforming, antenna integration technology, low-loss materials and fabrication

## Abstract

With the rapid evolution of 5G wireless communications, millimeter-wave (mmWave) technology has become a crucial enabler for high-speed, low-latency, and large-scale connectivity. As the critical interface for signal transmission, mmWave antennas directly affect system performance, reliability, and application scope. This paper reviews the current state of mmWave antenna technologies in 5G systems, focusing on antenna types, design considerations, and integration strategies. We discuss how the multiple-input multiple-output (MIMO) architectures and advanced beamforming techniques enhance system capacity and link robustness. State-of-the-art integration methods, such as antenna-in-package (AiP) and chip-level integration, are examined for their importance in achieving compact and high-performance mmWave systems. Material selection and fabrication technologies—including low-loss substrates like polytetrafluoroethylene (PTFE), hydrocarbon-based materials, liquid crystal polymer (LCP), and microwave dielectric ceramics, as well as emerging processes such as low-temperature co-fired ceramics (LTCC), 3D printing, and micro-electro-mechanical systems (MEMS)—are also analyzed. Key challenges include propagation path limitations, power consumption and thermal management in highly integrated systems, cost–performance trade-offs for mass production, and interoperability standardization across vendors. Finally, we outline future research directions, including intelligent beam management, reconfigurable antennas, AI-driven designs, and hybrid mmWave–sub-6 GHz systems, highlighting the vital role of mmWave antennas in shaping next-generation wireless networks.

## 1. Introduction

The rapid development of wireless communication technologies, coupled with the increasing prevalence of smart devices, the emergence of novel multimedia applications, and the widespread deployment of Internet of Things (IoT) systems, has led to an unprecedented surge in data traffic [1,2]. This trend necessitates the development of communication technologies that offer low latency and high reliability to support next-generation applications. For example, 5G wireless networks have been designed to address many of the limitations inherent in 4G systems, particularly from an architectural perspective. Moreover, 5G aims to support innovative services that exceed the technical capabilities and capacity requirements of existing networks, thereby revolutionizing key performance metrics such as data rates, latency, massive connectivity, network reliability, and energy efficiency [3]. These enhancements include ultra-low latency (as low as 1 ms) with ultra-high reliability, peak data rates reaching up to 10 Gbps—and potentially as high as 20 Gbps in certain scenarios—as well as a guaranteed minimum data rate of 100 Mbps. Furthermore, 5G is designed to achieve approximately 100 times greater energy efficiency and three times higher spectral efficiency compared to 4G networks [4].

As 5G technology has evolved, the enormous volume of data traffic and the escalating demand for higher communication capacity and signal integrity have led to the near-total occupation of frequency bands ranging from several hundred MHz to several GHz [5]. To meet these growing demands for increased data rates, two primary approaches are typically considered: transitioning to higher frequency bands to expand bandwidth, or enhancing spectral efficiency through the use of advanced modulation schemes or multiplexing techniques such as MIMO technology. Millimeter-wave (mmWave), with wavelengths between 1 mm and 10 mm, corresponds to a spectrum spanning 30 GHz to 300 GHz. Although the physical definition of mmWave strictly refers to the 30 GHz-300 GHz band (wavelength 1–10 mm), the 20 GHz band is often included in the mmWave category in practical applications. The communications industry, such as 3GPP, uniformly categorizes the frequency bands above 24 GHz as 5G mmWave (FR2 band), for example, 24.25–27.5 GHz (n258) and 26.5–29.5 GHz (n257). mmWave has emerged as a critical candidate for 5G wireless systems due to their broad bandwidth and high transmission rates [6,7]. These attributes make mmWave well-suited to fulfill future capacity needs. Currently, mmWave technology is being utilized in various applications, including radar systems (such as automotive radars), military communications, satellite communications, and point-to-point (P2P) communications. Moreover, the adoption of mmWave in 5G networks not only promises to address the increasing bandwidth requirements but also paves the way for innovative services and applications that require ultra-high reliability and low-latency connectivity. This makes mmWave indispensable for realizing the full potential of 5G technology across different sectors.

**Figure 1:**
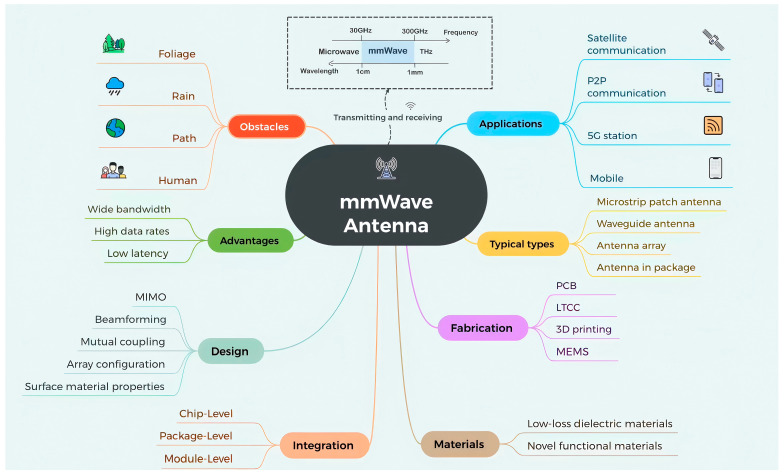
Overview of obstacles, advantages, design, integration, materials, fabrication, typical types, and applications of mmWave antennas.

Antennas, as essential components for transmitting and receiving electromagnetic signals, play a critical role in wireless communication and sensing systems [8]. In the context of mmWave communications, antennas are particularly vital due to the unique propagation characteristics and challenges associated with this high-frequency spectrum. For instance, mmWave signals are highly susceptible to blockage and attenuation from various environmental factors. Rain attenuation is one such challenge—since raindrops are typically on the order of a few millimeters, they can significantly obstruct mmWave signals, whose wavelengths are similarly sized [9]. Other common sources of signal degradation include foliage loss, human body blockage, and material penetration losses. Given these inherent limitations—such as high path loss, limited diffraction capability, and sensitivity to physical obstructions—advanced antenna designs and robust beamforming/beam tracking techniques are required to ensure reliable mmWave communication links [10]. Antenna arrays have emerged as a preferred solution in mmWave systems, enabling the generation of highly directional and steerable beams, thereby improving gain, directivity, and overall link efficiency [11]. Figure 1 presents the obstacles, pros, and design of mmWave antennas and their applications. Figure 1 presents the obstacles, advantages, design, integration, materials, fabrication, typical types, and applications of mmWave antennas.

Against this backdrop, this review article systematically explores the development and challenges of mmWave antennas within the context of 5G wireless communications. We examine key aspects including the types of mmWave antennas, MIMO-based array architectures, performance-influencing factors, integration technologies, as well as materials and fabrication techniques. The structure of this review is organized as follows:

Section 2 provides the methodology for writing this review.

Section 3 introduces the commonly used types of mmWave antennas and their performance characteristics.

Section 4 discusses design considerations for mmWave antennas, with a focus on MIMO architectures and key influencing factors.

Section 5 reviews state-of-the-art antenna integration technologies.

Section 6 presents an analysis of materials and fabrication techniques used in mmWave antenna manufacturing.

Finally, we summarize the current technical challenges and outline future research directions in this rapidly evolving field.

## 2. Methodology

In this review, we take a systematic approach for a comprehensive and unbiased selection of the relevant work on recent advancements in mmWave antennas for 5G applications. We mainly used Web of Science (WoS) supplemented by IEEE Xplore for conducting a literature search. To ensure the inclusion of the most relevant, highly cited, and up-to-date research, we prioritized peer-reviewed journal articles, review papers, and conference papers published between 2013 and 2025. This time frame was chosen to capture both foundational developments and the latest breakthroughs in the field.

### 2.1. Databases Searched

The selection of databases is critical for a comprehensive review. This study utilized WoS and IEEE Xplore due to their extensive coverage of peer-reviewed research in electrical engineering, wireless communication, and antenna technologies. WoS provides access to influential multidisciplinary journals, while IEEE Xplore offers a wealth of resources—including journal articles, conference papers, and standards—specifically relevant to mmWave antennas, MIMO systems, and 5G/6G technologies.

### 2.2. Search Keywords and Boolean Operators

The following keywords were used: mmWave antenna; mmWave MIMO; mmWave decoupling technology; mmWave antenna integration; mmWave materials; mmWave antenna fabrication. Boolean operators were also applied to search the following:

- mmWave antenna OR mmWave MIMO OR mmWave decoupling technology OR mmWave antenna integration OR mmWave materials OR mmWave antenna fabrication.
- mmWave antenna AND mmWave MIMO.

### 2.3. Inclusion and Exclusion Criteria

We gave priority to the papers that study millimeter wave frequency band and 5G requirements. For reference review articles, we selected the classic papers with high citations. Articles that focused on the design, fabrication, materials, and future trends of mmWave antennas, MIMO, and beamforming techniques were taken into account.

Inclusion Criteria:

- Articles that addressed performance evaluation, challenges, or future trends.
- Studies published between 2013 and 2025.
- Articles focused on design, analysis, fabrication, and materials.
- Classic review papers with high citations (without time window).

Exclusion Criteria:

- Non-English articles.
- Duplicate studies or articles lacking full-text access.
- Research on devices other than antennas.
- Dissertation or Thesis.
- Articles with similar content but published earlier.
- Articles with only abstract.

### 2.4. Literature Screening Process

**Figure 2:**
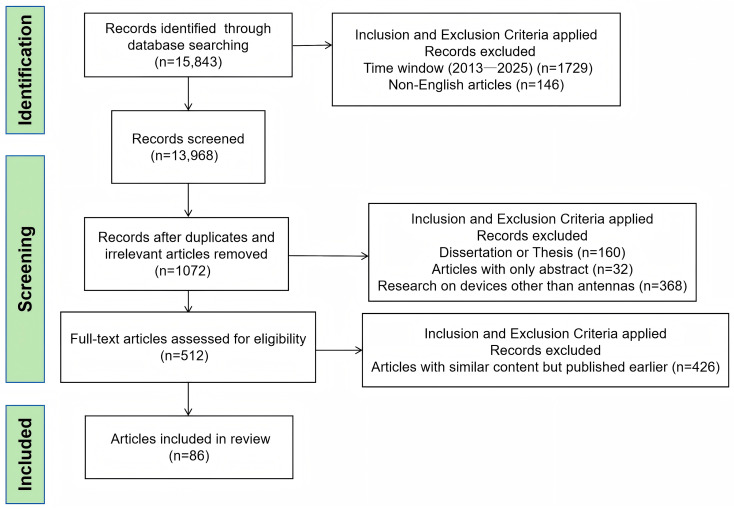
Systematic selection of articles.

After screening, a total of 86 articles were included in the references of this review. Figure 2 presents a summary of the literature screening process, illustrating the number of records identified, screened, and ultimately included in the final analysis.

## 3. Typical Types of Millimeter-Wave Antennas

Over the years, several antenna types have emerged as dominant solutions for mmWave applications, each with its own set of advantages, limitations, and suitable use cases. These antennas have been developed and refined in response to the unique challenges posed by mmWave propagation characteristics, such as high free-space path loss, limited penetration through obstacles, and sensitivity to environmental blockage.

### 3.1. Microstrip Patch Antennas

The microstrip patch antenna was first introduced in the 1970s and is characterized by its use of microstrip lines or coaxial probes to excite the radiating element. Owing to its simple structure, ease of fabrication, and relatively low manufacturing cost, it has been widely adopted in a variety of wireless communication systems [12,13]. With the rapid advancement of mmWave technologies, the compact size and high integration potential of microstrip patch antennas have made them particularly suitable for mmWave applications. Typical implementations include antennas for 5G mobile devices, automotive radar systems for collision avoidance, and Wi-Fi 6E communication modules.

Despite these advantages, microstrip patch antennas also exhibit several inherent limitations that constrain their performance in high-frequency regimes. These include a narrow operational bandwidth, which limits their effectiveness in mmWave scenarios requiring wideband operation; relatively low gain and limited power-handling capability, which restrict their applicability in high-performance systems; and significant mutual coupling in multi-element arrays, often necessitating additional decoupling structures or isolation techniques. Collectively, these challenges represent critical technical barriers that must be addressed to enable further performance enhancement and broader deployment of microstrip patch antennas in next-generation mmWave systems.

**Figure 3:**
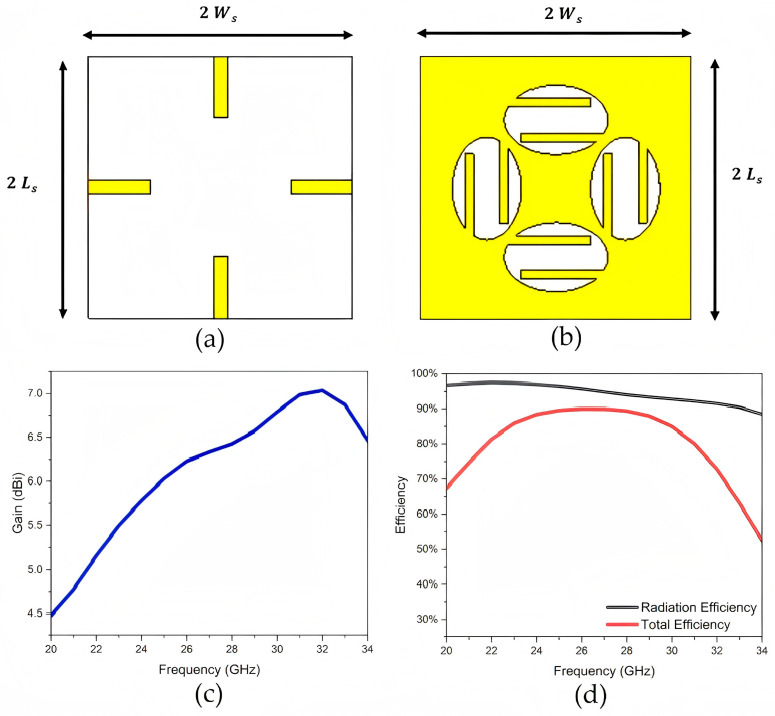
Compact elliptical slot mmWave MIMO antenna: (**a**) front view of antenna section, (**b**) back view of feeding section, (**c**) antenna gain, and (**d**) antenna efficiencies [15].

To overcome some of these limitations, various structural modifications have been proposed. For example, etching slots into the radiating patch has been shown to improve performance by enabling multimode resonance. The work in [14] describes a symmetric E-shaped patch antenna operating around the Ka-band, formed by introducing a longitudinal slot and two transverse slots on a rectangular patch. The fabricated prototype achieves an impedance bandwidth of 45.4%, with a peak gain of 8.5 dB across the entire operating band and a measured 3 dB gain bandwidth exceeding 30%. Figure 3 presents a compact elliptical slot four-element planar MIMO antenna designed with polarization diversity for mmWave operation in 5G systems, supporting a wide operating bandwidth of 9 GHz (22.2–31.4 GHz) at a −10 dB threshold, demonstrating a maximum gain of 6 dBi and an impressive radiation efficiency of 94%. The antenna’s behavior can be readily adjusted by altering the length of the metallic strips integrated into the elliptical slot [15].

**Figure 4:**
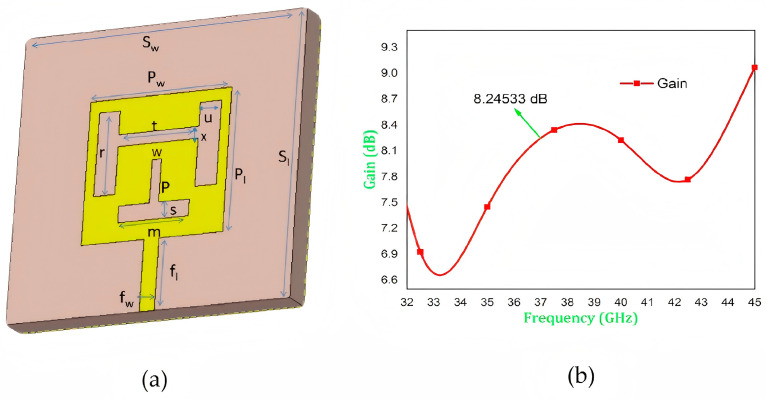
(**a**) Structure of the proposed antenna and (**b**) the gain of the antenna [16].

A square-slot microstrip patch antenna is presented in Figure 4 for mmWave communication at a resonant frequency of 37 GHz [16]. The design incorporates an H-shaped slot on the top side and an inverted T-shaped slot on the bottom of the radiating patch. This optimized structure enhances both the impedance bandwidth and gain. Simulation results show a return loss of −43.05 dB, a gain of 8.245 dB, and an impedance bandwidth of 16.22% at the resonant frequency, as illustrated in Figure 3b.

In addition to slot-based designs, several studies have explored structural and feeding optimizations to enhance performance. For instance, multilayer configurations with electromagnetic coupling feeds and grid arrays have been proposed to improve gain and bandwidth range [17]; coplanar parasitic elements have been employed to enhance the front-to-back ratio and power-handling capability [18]; and out-of-phase patch arrangements have been utilized to reduce mutual coupling and improve radiation pattern symmetry [19].

A performance comparison of typical mmWave microstrip patch antennas is shown in Table 1. As can be seen from Table 1, most microstrip patch antennas operate at the low-frequency band of mmWave and have the advantage of small size. In addition, the Rogers RT5880 series and Taconic TLY series are popular for the substrate type, showing their good characteristics. Although multi-layer antennas extend the operating frequency band of the antenna, the assembly complexity and interlayer effects are also not negligible. It is expected that the subsequent research on the microstrip patch antenna will make progress on the high-frequency band of mmWave and structure optimization in the future.

### 3.2. Waveguide Antenna

The waveguide antenna is a classical antenna structure that has been widely used in high-performance applications where microstrip patch antennas are less suitable. Compared with microstrip patch antennas, waveguide antennas offer higher power-handling capacity and radiation efficiency, better gain performance, and wider operational bandwidth. With the advancement of mmWave technologies, it has regained significant attention due to its high directivity, excellent radiation efficiency, and low loss characteristics. A typical waveguide antenna consists of three main components: the waveguide body (usually rectangular, circular, or multi-mode), the radiating aperture (e.g., horn-shaped, slot-type, or open-ended), and the feeding structure, which often involves a transition from coaxial or microstrip lines to the waveguide input. The key advantages of waveguide antennas lie in their ability to deliver high gain and strong directional radiation patterns, low insertion loss, and robust mechanical stability, making them well-suited for operation in harsh environments and broadband scenarios. Representative applications include mmWave base station antennas in 5G systems, terahertz (THz) imaging systems employed in medical diagnostics and security screening, and spaceborne communication front-ends operating in the Ka-band and beyond. However, waveguide antennas also face several limitations, including relatively large physical dimensions that hinder miniaturization, high manufacturing complexity leading to increased costs, and integration challenges when interfacing directly with radio frequency (RF) chips, thereby limiting their applicability in compact and highly integrated wireless devices.

To address these challenges, recent studies have proposed various innovative solutions. In order to optimize the antenna structure to reduce the manufacturing complexity and cost, Moschner et al. adopted a novel double-dipole waveguide feed structure based on low-cost additive manufacturing technology, enabling dual-polarized radiation and supporting polarization diversity or MIMO functionality. This approach overcame the limitations of traditional mmWave antennas in terms of cost, fabrication complexity, and performance [20]. In addition, a directional horn antenna was proposed, which adopted the substrate-integrated waveguide (SIW) technology that is superior to microstrip and conventional waveguides in the mmWave region, dual-element arrays, and extended structures to improve the antenna structure and achieve good directional characteristics [21].

Building on leaky-wave concepts, similarly, work in [22] utilized SIW technology for a novel reconfigurable H-plane horn leaky-wave MIMO antenna. This design incorporated dielectric loading and metamaterial structure arrays to boost gain, achieving 7.4 dBi at 24.99 GHz and 8.1 dBi at 26.1 GHz for its 4 × 4 MIMO configuration. A shared-aperture 2D leaky-wave antenna array with polarization and radiation beam reconfigurability was presented in [23]. This design eliminated open-circuit band-stopping through a novel cell featuring two asymmetric slots, attaining a peak gain of 23.6 dBi and 56.6% bandwidth (19–34 GHz).

For high-gain and low-loss array applications, the study [24] presented the design of a high-gain 16  ×  16-slot antenna array. Ridge gap waveguide technology was used to reduce the feeding network loss and achieve a low-loss array antenna. The feed layer of the proposed antenna was coupled to a standard rectangular waveguide (WR-28) using a proper transition. The measured results showed an impedance bandwidth of more than 17% over the frequency range of 27.5–32.6 GHz, a maximum gain of 28.9 dBi, and SLL lower than − 20 dB. Zhang et al. adopted the design of all-metal planar array antenna to avoid the serious dielectric loss of mmWave and the combination of ridge gap waveguide (RGW) and E-plane groove gap waveguide (E-GGW) radiation elements to improve the bandwidth [25]. A maximum gain of 27.7 dBi was obtained, with an impedance matching bandwidth of 46.8% during the 18.8 to 30.3 GHz period.

Table 2 shows the research results of several mmWave waveguide antennas, highlighting key performance metrics and design configurations reported in recent years. Among various waveguide technologies, SIW-based antennas have gained increasing popularity and are moving toward mainstream adoption due to high performance and cost-effectiveness. However, the size of SIW-based antennas becomes more challenging at higher mmWave frequencies, where miniaturization is critical. Manufacturing complexity also increases due to tighter tolerances and precision requirements in fabrication. The optimization of waveguide antenna performance therefore demands innovative structural designs. Continued progress will require joint efforts from researchers to address these technical challenges.

### 3.3. Antenna Array

While antenna arrays have been mentioned in previous sections, this section provides a more detailed and systematic overview of their architecture, functionality, and role in mmWave communications. An antenna array consists of multiple radiating elements arranged in a predefined configuration, where the phase and amplitude of each element are controlled to achieve beamforming. This capability enables enhanced signal strength, improved spatial directionality, and dynamic adaptation to channel conditions, making antenna arrays a cornerstone technology for mmWave communications [26,27]. With the rise of large-scale MIMO systems, antenna arrays have become essential for realizing high-data-rate and low-latency wireless links in next-generation networks. The fundamental components of an antenna array include individual radiating elements—such as patch, waveguide, or Vivaldi antennas [28]—a feeding network (e.g., Butler matrices or T/R modules), a phase-shifting mechanism for beam steering, and a packaging structure that ensures mechanical protection and system integration. Key advantages of antenna arrays include their ability to provide high gain and directional radiation patterns, support intelligent beam management, improve spectral efficiency through spatial multiplexing, and meet the stringent requirements of enhanced mobile broadband (eMBB) and ultra-reliable low-latency communication (URLLC) scenarios. Notable applications include mmWave user equipment (UE), such as the external mmWave antenna module in the Motorola Moto Z3 smartphone; active antenna units (AAUs) in mmWave base stations from vendors like Nokia AirScale and ZTE; and airborne communication systems for high-speed unmanned aerial vehicles (UAVs), where stable mmWave links are critical. Despite their performance benefits, antenna arrays face several technical and practical challenges, including increased system complexity and power consumption, mutual coupling between closely spaced elements that degrades isolation, complicated calibration and synchronization mechanisms, and high manufacturing and deployment costs, which hinder their widespread adoption in cost-sensitive and compact devices. The following describes several promising solutions proposed in recent years.

**Figure 5:**
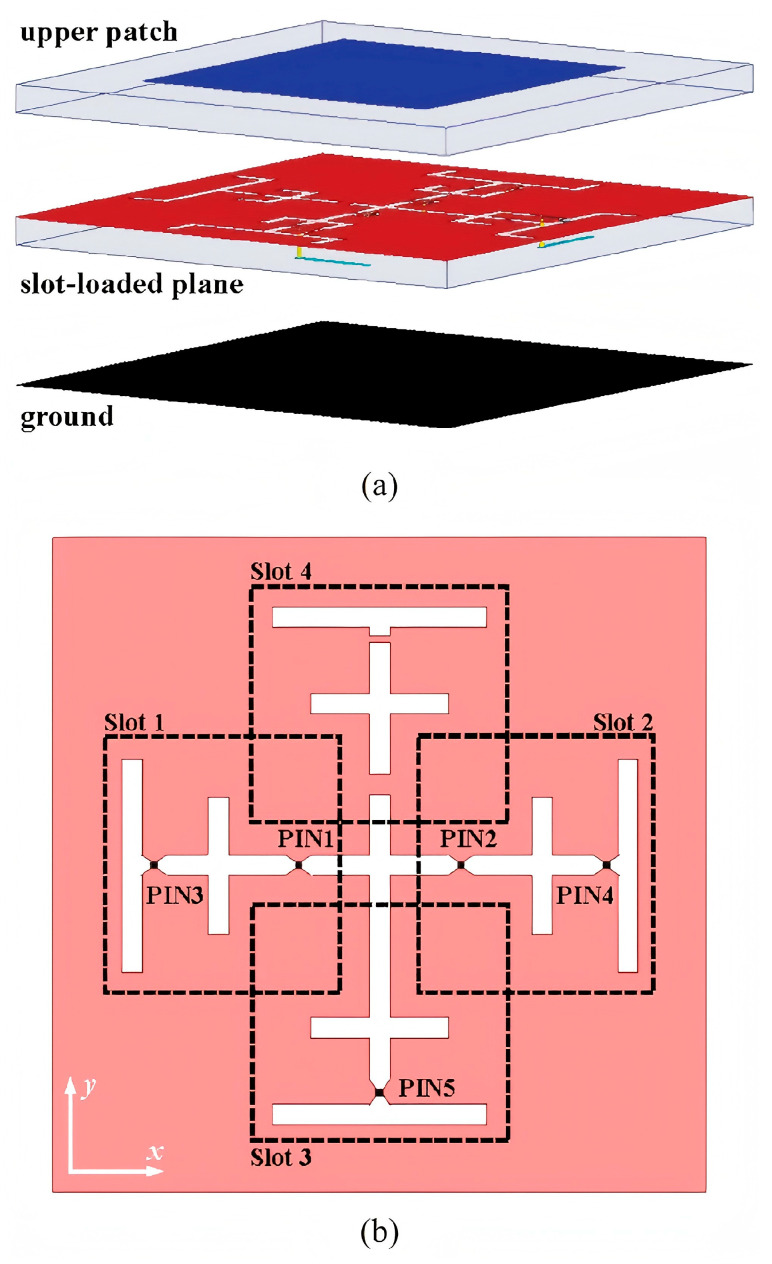
Structure of the proposed 2-bit RIS element: (**a**) exploded view; (**b**) detailed view of the slot-loaded plane [29].

In 2020, Dai et al. proposed a novel reconfigurable intelligent surface (RIS) comprising 256 2-bit elements (Figure 5), which integrated both phase-shifting and radiation functionalities on a single electromagnetic surface. Operating at 28.5 GHz, the design achieved an antenna gain of 19.1 dB while significantly reducing power consumption compared to conventional phased array antennas [29].

A low-cost antenna array utilizing 3D-printed dielectric polarizers was proposed, in which the entire structure was implemented on a single substrate [30]. This design significantly reduced manufacturing complexity and cost. Furthermore, the adoption of a microstrip line (MSL) feeding mechanism facilitated seamless integration with transceiver circuits, offering a distinct advantage over other waveguide-fed antennas. Table 3 shows the performance comparison of the above antenna arrays.

In terms of array antenna beamforming synchronization and calibration in time and frequency domains, several algorithmic improvements have been introduced. For instance, a synchronization framework for hybrid mmWave MIMO systems was proposed [31], and the Swift-Link algorithm was also described [32], both aiming to enhance system stability and performance under practical operating conditions. Solutions of beam management based on artificial intelligence (AI) and machine learning (ML) frameworks have also garnered significant attention, particularly in applications such as beam prediction and beam tracking [33]. An AI/ML model can intelligently predict optimal beam directions by analyzing historical channel state information, user behavior patterns, or environmental sensing data, which is very promising in dealing with complex beam situations of large antenna arrays. However, the computational and resource costs of model training remain a notable concern.

### 3.4. Antenna-in-Package (AiP)

AiP technology integrates antenna elements directly with RF front-end circuits within a single package, offering a compact and system-level solution for mmWave communication systems. This approach addresses the increasing demand for miniaturization, high integration, and cost-effective manufacturing in next-generation wireless devices. An AiP antenna typically consists of radiating elements—often based on patch, slot, or dipole configurations—substrate materials such as organic laminates, LTCC, or fan-out wafer-level packaging (FOWLP), and embedded RF components including phase shifters, power amplifiers, and beamforming ICs. One of the key advantages of AiP antennas is their ability to minimize interconnect losses between the antenna and transceiver, thereby improving overall system efficiency. Additionally, they support multi-band and multi-polarization operations while enabling 3D packaging and heterogeneous integration. These features make AiP antennas particularly suitable for mobile and wearable applications where space is limited but performance requirements are high. Representative commercial implementations include Qualcomm’s QTM052 mmWave antenna module for 5G smartphones, Intel’s AiP solutions in early 5G laptop modems, and Samsung’s phased-array-based AiP modules used in automotive radar and short-range communication systems. Despite these benefits, AiP technology faces several challenges, including thermal management due to the close proximity of active components, limitations in radiation efficiency caused by substrate losses, and difficulties in achieving wide bandwidth and high gain simultaneously. Moreover, standardization and cost-efficient mass production remain critical issues that must be addressed to enable broader deployment across consumer electronics and industrial applications.

Embedded wafer-level ball grid array (eWLB) packaging offers a cost-effective solution for the mass production of AiP [34]. High-density interconnect (HDI) technology provides an alternative for low-cost, large-scale mmWave AiP fabrication. It supports the integration of multiple industry-standard dielectrics with layer thicknesses from 10 to 100 μm, enabling design flexibility and heterogeneous integration. An example is the integrated passive device (IPD) antenna based on a multilayer HDI printed circuit board (PCB) structure presented in [35]. Table 4 presents three processes applied to AiP manufacturing.

In summary, each type of millimeter-wave antenna possesses distinct advantages and inherent limitations, making the selection of an appropriate antenna design critical for specific application scenarios. Future research should focus on technological innovation and improved cost-effectiveness to enable the broader adoption of mmWave communication technologies in consumer electronics and industrial applications. Moreover, interdisciplinary collaboration will be essential to overcoming existing technical bottlenecks and exploring novel solutions.

## 4. Millimeter-Wave Antenna Design: The Application of MIMO Technology and Factors Related to Antenna Gain and Efficiency

In wireless communication systems operating in the mmWave band, traditional single-antenna systems struggle to meet the demands for high data rates and reliable connectivity due to significant path loss. As a result, MIMO technology has been widely adopted in mmWave antenna design to enhance system capacity and link performance through techniques such as spatial multiplexing, beamforming, and spatial diversity. At the same time, to achieve high-performance antenna arrays within limited space, it is essential to carefully consider key factors that affect antenna gain and efficiency, including beamforming strategies, mutual coupling effects, array configuration, and surface material properties.

### 4.1. Application of MIMO Technology in Millimeter-Wave Antenna Systems

Millimeter-wave bands have attracted increasing attention due to their abundant bandwidth resources. However, the inherent increase in free-space path loss at these high frequencies presents new challenges for antenna design. According to Friis transmission equation [36],

(1) $$P_{r}=G_{r}G_{t}{\left(\frac{\lambda }{4\pi d}\right)}^{2}P_{t}$$

where the powers are in linear scale, *d* is the TX-RX separation distance, *λ* is the wavelength and *G_t_* and *G_r_* are the transmit and receive antenna gains, *P_t_* is the transmitted power. The received power *P_r_* decreases with the square of the wavelength *λ*, meaning that mmWave signals suffer significantly more attenuation than lower-frequency signals in the absence of directional gain compensation. To address these challenges, MIMO technology has become a key enabler in mmWave antenna design. By leveraging spatial multiplexing, beamforming, and diversity gains, MIMO overcomes the limitations of single-antenna systems.

**Figure 6:**
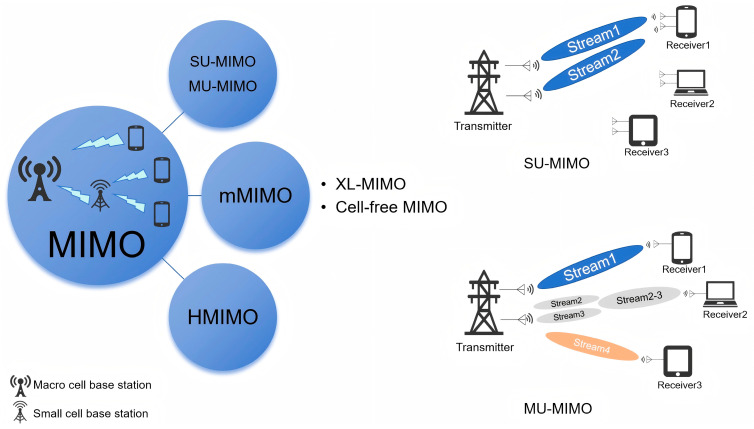
The division of MIMO systems.

Spatial multiplexing enables the simultaneous transmission of multiple independent data streams, thereby increasing data rates. Spatial diversity improves link reliability by exploiting signal replicas over different propagation paths, reducing the bit error rate without increasing bandwidth or transmit power. Beamforming precisely controls the phase and amplitude of each antenna element to focus signal energy in specific directions, compensating for the high path loss characteristic of mmWave signals and reducing interference [37]. By using multiple transmitting and receiving antennas to simultaneously transmit multiple data streams, MIMO significantly enhances data rates and channel capacity. This technology not only increases system throughput but also improves link quality and reliability [38]. With technological advancements, MIMO has evolved into massive MIMO, significantly enhancing spatial multiplexing gains and improving link robustness. MIMO systems can be broadly categorized into four types, as demonstrated in Figure 6, each tailored to specific application scenarios and performance objectives.

#### 4.1.1. Single-User MIMO (SU-MIMO)

SU-MIMO improves spectral efficiency by deploying multiple antennas at both the transmitter and receiver ends of a single user device. It utilizes spatial multiplexing to split data streams into multiple independent streams transmitted simultaneously through different antennas. Spatial diversity is also employed to enhance link reliability without increasing bandwidth or transmit power. At the receiver, multiple antennas are used to separate and reconstruct the original data streams.

#### 4.1.2. Multi-User MIMO (MU-MIMO)

In MU-MIMO systems, a multi-antenna base station serves multiple single-antenna users concurrently. This configuration shifts hardware complexity to the base station side, allowing cost-effective single-antenna terminals while still achieving multiplexing gain. Additionally, MU-MIMO benefits from multi-user diversity, making it more robust than point-to-point SU-MIMO systems in environments with poor scattering conditions [39].

#### 4.1.3. Massive MIMO (mMIMO)

Massive MIMO employs large-scale antenna arrays at the base station to serve multiple users simultaneously, offering significant improvements in spectral efficiency, energy efficiency, and throughput [40,41,42]. A typical mMIMO system may involve hundreds of antennas serving dozens of users, with each user able to reduce its transmit power proportionally based on the number of base station antennas [39]. mMIMO has become a cornerstone of 5G and is evolving towards extremely large MIMO (XL-MIMO) and cell-free mMIMO architectures in preparation for 6G deployments.

The basic idea of XL-MIMO is to deploy an extremely large number of antennas in a compact space. Compared with mMIMO, XL-MIMO has the following characteristics: more flexible hardware designs, a much larger number of antennas, the much smaller antenna spacing, new EM characteristics, near-field based signal processing, which can provide stronger beamforming gain, richer degrees of freedom (DoF), and spectral efficiency (SE) [43].

Since users at cell boundaries may suffer from strong inter-cell interference, the cell-free MIMO system removes the concept of cell boundaries. By deploying a large number of geographically distributed access points (APs) connected to a central processing unit (CPU), cell-free MIMO can effectively address the inter-cell interference that exists in the intrinsic implementation of a “cell-centric” network [44].

The two technological approaches obtain convenience, but bring greater power consumption, computational complexity, and channel security problems, which necessitate algorithmic and hardware enhancements to meet the above challenges.

#### 4.1.4. Holographic MIMO (HMIMO)

Emerging from advances in metamaterials and RIS, HMIMO represents a paradigm shift by transforming wireless environments into programmable entities. mMIMO uses discrete deployed antennas to form a discrete array aperture, while HMIMO unit elements are placed more and more densely to form an almost spatially continuous aperture, making them capable of forming very sharp beams with weak sidelobes. Interestingly, the mutual coupling effect, which is considered harmful in traditional communication systems, can be used appropriately in HMIMO surfaces to achieve super-directionality [45,46]. Utilizing holographic modulation and continuous aperture surfaces, HMIMO aims to achieve unprecedented spectral efficiency and spatial resolution while maintaining low hardware complexity [47]. It holds great promise for future THz communications, dense IoT networks, and smart radio environments, although it remains in early research stages with many technical challenges yet to be resolved such as complex channel estimation, resource allocation, and beamforming; therefore, there are no unified technical standards and mature industrial ecology, and there is still a long way to go before large-scale commercial deployment.

### 4.2. Key Factors Affecting Antenna Gain and Efficiency in Millimeter-Wave Systems

The performance of mmWave antennas is influenced by a range of critical factors, including beamforming strategies, mutual coupling effects, array configuration, and surface material properties. These elements directly impact key antenna metrics such as radiation efficiency, directional stability, and overall communication quality. Given the high-frequency nature of mmWave systems and the stringent performance requirements, the influence of these factors becomes even more pronounced. To address these challenges, various optimization approaches and decoupling techniques have been developed and widely adopted. These methods aim to enhance antenna gain, improve beam steering accuracy, and effectively suppress electromagnetic interference between array elements, thereby ensuring reliable and efficient operation in mmWave communication systems.

#### 4.2.1. Beamforming Strategies

Beamforming is considered as a key enabling technique for mmWave band communications [48], which is usually achieved by directing the transmitted signal towards the receiver while suppressing it in a direction other than that of the intended receiver in order to provide significant array gain, providing a higher signal-to-noise ratio (SNR) and additional radio link margin, thus mitigating the propagation path loss, which can be accomplished using digital, analog, or a combination of digital and analog beamforming techniques [37].

Digital beamforming (DBF) relies on pre-processing the transmitted signal in the digital domain and then post-processing the received signal at the receiver [49]. However, digital beamforming adds an extra cost at high frequencies because each antenna requires its own analog RF front-end chain, which results in larger transceivers and higher power consumption.

Analog beamforming is a simple but effective method to bend and control electromagnetic waves without the need for digital signal processing. The technique uses a series of analog devices, including phase shifters and amplifiers, to change the phase and amplitude of the signal in real time to ensure that the signal is transmitted or received in a certain direction or at a specified destination [50], and can produce high beamforming gains from a large number of antennas, but is not as flexible as digital beamforming, and it is this trade-off between flexibility/performance and simplicity that is driving the need for hybrid beamforming architectures, especially when large numbers of antennas are required, such as in the mmWave band [51].

Hybrid beamforming allows the transmitted signal to be processed first through a phase shifter in the digital domain, without the need for an RF chain. As a result, the dimensionality of the signal can be greatly reduced. The post-processed signals undergo conventional analog beamforming to build mmWave MIMO systems with much lower complexity. At the heart of hybrid beamforming is the division of precoding between the analog and digital domains, which allows for an effective trade-off between low-complexity but limited performance analog beamforming and high-complexity, high-performance all-digital precoding [52,53].

#### 4.2.2. Mutual Coupling Effects

In compact MIMO antenna designs, mutual coupling is a significant challenge. To meet miniaturization requirements, the spacing between antenna elements is often reduced, leading to increased electromagnetic coupling, which affects impedance matching and radiation efficiency [54,55]. This effect is even more pronounced in the mmWave band due to shorter wavelengths. To mitigate this issue, various decoupling techniques have been proposed, such as defective ground structures (DGSs) [56], parasitic element decoupling techniques (PDTs) [57], slit structures [58], dielectric resonator antennas (DRAs), vias [59], complementary split-ring resonators (CSRRs) [60], and electromagnetic bandgap (EBG) structures [61]. These techniques aim to provide high isolation while reducing antenna size, thereby improving overall antenna performance. The performance of different decoupling techniques is shown in Table 5.

#### 4.2.3. Array Configuration

The configuration of an antenna array significantly impacts the performance of mmWave antennas. Different array configurations (such as rectangular, cross-shaped, circular, and hexagonal arrangements) affect directionality, gain, and main lobe width. For example, circular arrays, due to their larger area and uniform element spacing, offer higher array gain and directivity. In terms of beam alignment, circular arrays have wider main lobes, resulting in less performance loss when the beam is not perfectly aligned. In contrast, narrow beams in rectangular and cross-shaped arrays are more susceptible to antenna vibration or external environmental factors, increasing the likelihood of communication disruptions [10]. Therefore, carefully selecting and designing array configurations is crucial for optimizing the performance of mmWave antennas.

#### 4.2.4. Surface Material Properties

**Figure 7:**
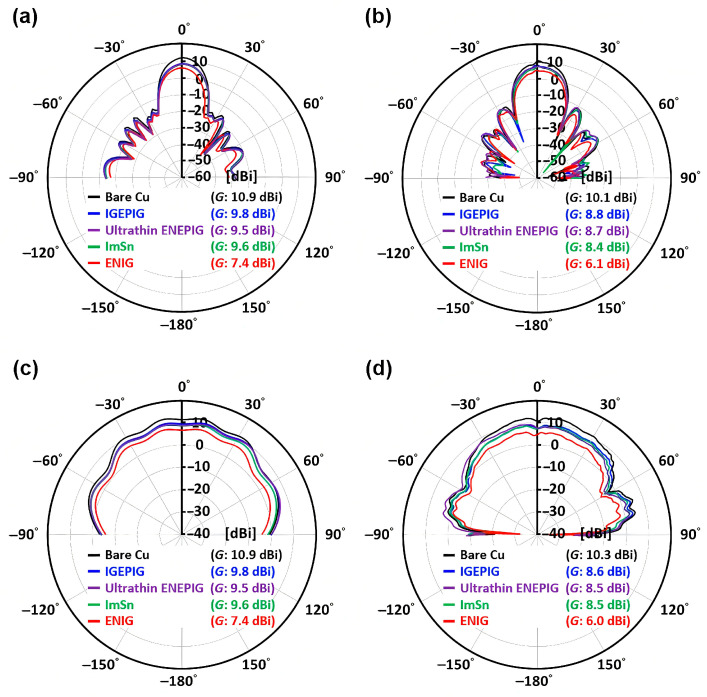
Two-dimensional radiation patterns of the comb-line antenna with different surface finishes at f = 77 GHz: (**a**) HFSS simulation (yz-plane); (**b**) anechoic chamber measurements (yz-plane); (**c**) HFSS simulation (xz-plane); (**d**) anechoic chamber measurements (xz-plane) [62].

The choice of surface coating materials also has a significant impact on the performance of mmWave antennas. Different surface coating materials (such as Au/Pd/Ni(P) (ENIG), Au/Pd/Ni(P) (ENEPIG), ultra-thin Ni(P)-type ENEPIG, immersion tin (ImSn), Au/Pd(P)/Au (IGEPIG), and immersion gold (IG)) affect the scattering parameters (S-parameters), radiation patterns, gain, and temperature distribution of the antenna. Studies [62] have shown that surface coatings containing thick Ni(P) layers can lead to center frequency shifts and gain degradation, whereas ultra-thin Ni(P)-type ENEPIG and IGEPIG coatings exhibit better antenna performance (Figure 7). Additionally, with the development of metamaterials and RIS, new possibilities for surface coating techniques based on these advanced materials are emerging, providing innovative solutions for mmWave antenna design.

In summary, MIMO technology serves as a cornerstone in mmWave antenna design, effectively addressing challenges such as high path loss and strong interference inherent in the mmWave band. It significantly enhances data transmission rates and channel capacity, laying a solid foundation for more efficient and reliable wireless communication systems. In parallel, factors including beamforming strategies, mutual coupling, array configuration, and surface material properties collectively determine the radiation efficiency, directivity, and overall performance of mmWave antennas. The synergistic integration of MIMO technology with these key antenna design considerations is not only essential for maximizing the potential of mmWave communications but also lays a solid technical foundation for the evolution towards 6G and future THz communication systems.

## 5. Integration Technologies for Millimeter-Wave Antennas

**Figure 8:**
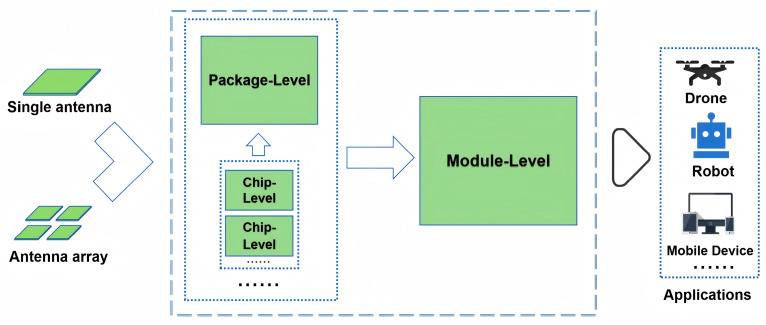
Three levels of integration technologies for mmWave antennas.

With the increasing demand for high data rates, low latency, and dense connectivity in 5G and future 6G communications, mmWave antenna systems are evolving towards highly integrated architectures [63,64]. This trend not only helps reduce device size and power consumption but also enhances overall system performance and deployability. Currently, the integration technologies of mmWave antennas can be categorized into three main levels: chip-level integration, package-level integration, and module-level integration. The relationship among the three levels is shown in Figure 8. These approaches offer complementary advantages across different application scenarios and together facilitate the commercialization of mmWave communication systems.

### 5.1. Chip-Level Integration

Chip-level integration refers to the co-design and co-fabrication of antenna elements with RF front-end circuits—such as low-noise amplifiers (LNAs), power amplifiers (PAs), and phase shifters—on the same substrate, or even within a single system-on-chip (SoC) architecture. This approach minimizes interconnect losses and parasitic effects that typically occur between discrete components, making it particularly suitable for compact system designs at high frequencies.

The key advantages include significantly reduced signal path length, which lowers transmission loss and improves efficiency, as well as enabling fine-grained beamforming control for enhanced directivity and stability. However, chip-level integration also presents several challenges, such as the high dielectric loss of silicon substrates at mmWave frequencies, thermal management issues, and potential mutual interference between the antenna and active circuits. As a result, researchers have been exploring alternative high-performance semiconductor materials—such as GaAs, SiGe, and GaN—to optimize the radiation efficiency and overall performance of on-chip antennas [65].

### 5.2. Package-Level Integration

Package-level integration, commonly implemented as AiP, represents a compromise between chip-level and module-level integration. In this approach, the antenna is embedded within the chip packaging structure, allowing it to coexist with the RF transceiver chip inside the same package. AiP strikes a balance between performance and cost-effectiveness, making it the dominant solution for mmWave applications in mobile devices.

By leveraging advanced packaging technologies—such as FOWLP, flip-chip bonding, and wafer-level packaging (WLP)—AiP enables the integration of multi-antenna arrays and RF front-ends within limited space. Its benefits include good electromagnetic compatibility, ease of mass production, and compatibility with standard CMOS processes. Additionally, AiP supports the design of multi-band and multi-polarization antennas, meeting the diverse requirements of 5G smartphones, AR/VR headsets, automotive radars, and other mobile platforms.

Despite its advantages, AiP faces certain limitations, such as dielectric losses from packaging materials and restricted antenna size that may compromise directivity. Therefore, ongoing research focuses on developing new packaging materials with low dielectric constants, 3D stacked structures, and heterogeneous integration techniques to further enhance AiP antenna performance [66].

### 5.3. Module-Level Integration

Module-level integration involves integrating the mmWave antenna array, RF front-end modules (RFFEs), beamforming chips, filters, and power management units into a single functional module. This approach retains a degree of flexibility and maintainability while achieving high system integration, making it widely used in base stations, access points, industrial robots, drones, and other semi-fixed or fixed deployment scenarios.

The advantages of module-level integration include rapid deployment through standardized interfaces, simplified system complexity, and improved reliability through optimized thermal and mechanical design. High-performance substrates such as LTCC, HDI PCB, and flexible PCB are often employed, along with metal shielding and cavity filters to achieve excellent electrical performance and interference suppression.

Moreover, module-level integration supports advanced architectures such as large-scale MIMO and hybrid beamforming, providing a viable technical platform for emerging technologies like RIS and distributed antenna systems (DASs) in 6G communications [67,68].

In conclusion, the integration technologies for mmWave antennas are evolving towards a multi-layered, synergistic development path. Chip-level integration emphasizes performance optimization and miniaturization, package-level integration prioritizes process compatibility and mass production feasibility, and module-level integration focuses on system scalability and engineering practicality. Table 6 summarizes the key differences among the three levels of integration technologies. Together, these integration strategies form a critical technical chain—from core components to full system deployment—for realizing mmWave communication systems. With advancements in advanced manufacturing processes, novel materials, and AI-assisted design tools, mmWave antenna integration technologies will continue to evolve, laying a solid foundation for the upcoming era of 6G and THz communications.

## 6. Fabrication and Material Selection for Millimeter-Wave Antennas

Millimeter-wave antennas operate in the high-frequency range of 30 GHz to 300 GHz, where signal wavelengths are short and manufacturing precision, material performance, and process compatibility are critically important. The choice of fabrication method and materials directly affects key performance metrics such as radiation efficiency, insertion loss, and directivity, and also determines the feasibility and stability of the antenna in practical communication systems. Currently, common fabrication techniques include PCB printing, LTCC, 3D printing, and MEMS processing, each with its own characteristics and suitable for different application scenarios. At the same time, the use of low-loss dielectric materials and novel functional materials has introduced new possibilities for mmWave antenna design.

### 6.1. Fabrication Technologies

#### 6.1.1. PCB Printing Technology

PCB printing is one of the most widely used methods for antenna fabrication, especially suitable for microstrip patch antenna designs. This technology is mature, cost-effective, and conducive to mass production, making it ideal for mid-to-low frequency applications. However, at mmWave frequencies, traditional FR4 substrates exhibit significant dielectric and conductor losses, leading to increased signal attenuation and limiting their use in high-performance mmWave systems. Therefore, replacing conventional materials with low-loss high-frequency substrates—such as PTFE, hydrocarbon-based materials, or LCP—is essential for improving the performance of PCB-based antennas.

#### 6.1.2. LTCC Technology

LTCC is a multilayer ceramic integration technique that offers excellent dielectric properties, thermal stability, and mechanical strength, making it well-suited for mmWave applications [69,70]. With LTCC, complex feed networks and antenna structures can be realized in three-dimensional space, effectively reducing interconnect losses and parasitic effects. Additionally, LTCC supports high-density integration, enabling the co-design of RF FEMs and antennas. It is widely used in mmWave radar, 5G base stations, and satellite communications.

#### 6.1.3. Three-Dimensional Printing Technology

With the development of additive manufacturing technologies, 3D printing has shown unique advantages in the fabrication of mmWave antennas. This technique enables the rapid prototyping of highly complex geometries, such as irregular shapes, curved surfaces, lens antennas, and metamaterial structures, surpassing the limitations of traditional fabrication methods [71,72]. Three-dimensional printing also offers flexible design iteration capabilities and relatively low development costs, making it particularly suitable for prototype verification and small-batch customized production. Currently, both metal 3D printing and polymer-based 3D printing combined with metal coating have been applied in mmWave antenna fabrication, with potential for further improvements in resolution and electrical performance.

#### 6.1.4. MEMS Technology

MEMS technology is used to fabricate miniature reconfigurable antennas capable of dynamically adjusting frequency, polarization, or beam direction [73]. These antennas are particularly suitable for smart beamforming and adaptive communication systems. MEMS switches and varactors can be embedded into the antenna structure to achieve functions such as beam steering and frequency switching. Although still in the developmental stage for mmWave applications, MEMS technology shows great potential in miniaturization, low power consumption, and multifunctional integration.

The key features and typical applications of these technologies are summarized in Table 7. In summary, manufacturing technologies for mmWave antennas are evolving from isolated, single-process approaches toward a holistic paradigm of “material–process–function” co-design. Future advancements will depend not only on improved fabrication precision, but increasingly on the integration of multi-scale manufacturing capabilities and intelligent functionalities. This convergence lays a robust foundation for next-generation 6G and THz communication systems.

### 6.2. Material Selection

#### 6.2.1. Low-Loss Dielectric Materials

Millimeter-wave antennas are highly sensitive to the dielectric constant (ε_r_) and loss tangent (tanδ) of the substrate materials. Commonly used low-loss high-frequency dielectric materials include the following:

- PTFE: A widely used high-frequency material due to its extremely low dielectric constant (ε_r_ ≈ 2.0–2.2) and loss tangent (tanδ ≈ 0.0004–0.001), making it ideal for microwave and mmWave applications. It is often reinforced with glass fibers or other fillers to improve mechanical rigidity. Its chemical inertness and thermal stability further ensure reliable performance under harsh conditions. It offers stable dielectric properties and good thermal management, making it widely used in mmWave antenna applications [74,75].
- Hydrocarbon-based materials: Cost-effective and process-friendly alternatives that combine hydrocarbon resins with ceramic fillers to achieve moderate dielectric constants (ε_r_ ≈ 3.0–4.0) and low loss tangent (tanδ ≈ 0.002–0.004). They offer good dimensional and thermal stability, and importantly, they can be manufactured using standard FR-4 processes, which significantly reduces production costs. Their electrical performance is comparable to that of PTFE-based materials.
- LCP: A thermoplastic material characterized by low dielectric loss (tanδ ≈ 0.002–0.004), excellent moisture resistance, and good thermal stability over a wide temperature range. Its flexibility and dimensional stability make it particularly suitable for flexible and wearable mmWave antenna designs [76,77].
- Microwave dielectric ceramics: Known for their superior electrical and mechanical properties, especially in high-frequency and high-power applications. These ceramics have a wide range of dielectric constants (ε_r_ ≈ 5–40) and extremely low loss tangents (tanδ ≈ 0.0001–0.001), along with high mechanical strength and thermal stability. This makes them ideal for components requiring precise frequency control and long-term reliability, such as filters, resonators, and antennas. Additionally, microwave dielectric ceramics are compatible with LTCC technology, enabling compact integration and widespread use in mmWave radar systems, 5G base stations, and satellite communication modules [78,79,80,81].

**Figure 9:**
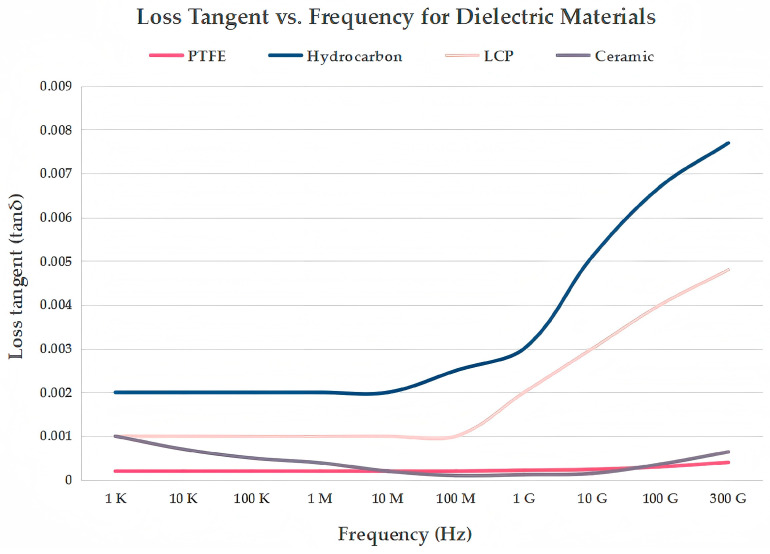
Schematic plot of loss tangent as a function of frequency for low-loss dielectric materials.

In fact, dielectric loss is highly frequency dependent. Figure 9 illustrates the variation of the loss tangent for PTFE, hydrocarbon-based materials, LCP, and microwave dielectric ceramics as a function of frequency. Therefore, the operating frequency must be carefully considered when selecting low-loss dielectric materials. The evolution of low-loss dielectric materials is shifting from isolated property optimization toward a system-level paradigm that emphasizes multifunctionality, process compatibility, and environmental robustness.

#### 6.2.2. Novel Functional Materials

In recent years, several emerging materials have brought breakthroughs to mmWave antenna design:

- Graphene: With ultra-high electron mobility and tunable electromagnetic response [82], graphene can be used in reconfigurable antennas, absorbers, and frequency selective surfaces (FSSs), enhancing antenna flexibility and performance.
- Metamaterials: Engineered sub-wavelength structures enable exotic electromagnetic properties such as negative refraction, perfect absorption, and anomalous reflection, helping realize compact, highly directive, and broadband mmWave antennas [83,84].
- Smart and Phase-Change Materials: Examples are VO_2_ (vanadium dioxide) and GST (Ge_2_Sb_2_Te_5_), which can switch between metallic and insulating states under external stimuli (e.g., current, light, temperature), enabling dynamically tunable antenna functions [85,86].

The selection of these materials should consider multiple factors, including cost, processability, environmental adaptability, and compatibility with system integration requirements. To achieve optimal performance in mmWave antenna design, careful material selection is essential. The representative examples of the above-mentioned materials and their key advantages are summarized in Table 8. Beyond conventional dielectrics, the emergence of novel functional materials is opening transformative pathways for next-generation mmWave antennas. While challenges remain in large-scale fabrication, long-term reliability, and integration complexity, these advanced materials represent a paradigm shift from passive to active, intelligent, and adaptive antenna systems. Their development not only expands the design space for mmWave antennas but also aligns closely with the vision of cognitive radio, smart environments, and 6G wireless networks, where responsiveness and multifunctionality are paramount.

In summary, the choice of fabrication technology and material selection plays a critical role in determining the performance of mmWave antennas. Regarding various fabrication techniques—such as PCB printing, LTCC, 3D printing, and MEMS—each offers distinct advantages, making it suitable for different application scenarios, including low-cost consumer devices, high-performance communication systems, prototyping of complex structures, and reconfigurable antenna designs, respectively. At the same time, the appropriate selection of low-loss dielectric and novel functional materials not only significantly improves the radiation efficiency and directivity of antennas but also drives the evolution of mmWave antennas towards greater intelligence, efficiency, and flexibility. Looking ahead, with continuous advancements in advanced manufacturing technologies and materials science, mmWave antennas will play an increasingly important role in cutting-edge fields such as 6G, THz communications, intelligent sensing, and the IoT.

## 7. Future Challenges and Development Prospects of Millimeter-Wave Antennas

Despite significant progress in mmWave antenna technology over the past decade, with promising applications in 5G communications, vehicular networking (V2X), industrial automation, augmented reality (AR/VR), and satellite communications, several key challenges remain before these systems can be widely deployed and commercialized. At the same time, with the rapid development of artificial intelligence, novel materials, and intelligent surfaces, mmWave antennas are expected to witness broad prospects for innovation and performance enhancement.

### 7.1. Propagation Path Limitations: Signal Blockage and Coverage Constraints

**Figure 10:**
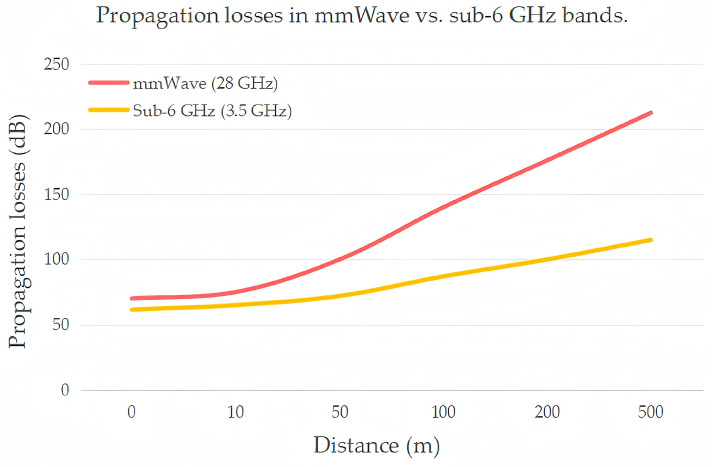
Schematic diagram of propagation losses for mmWave and sub-6 GHz.

Millimeter-wave signals operate at high frequencies with short wavelengths, offering abundant bandwidth but suffering from poor penetration and diffraction capabilities. Even small obstacles—such as human bodies, walls, or foliage—can cause severe signal attenuation or even complete link failure, limiting the coverage and reliability of mmWave communication systems. Figure 10 shows a schematic diagram of propagation losses for mmWave and sub-6 GHz as a function of distance.

To address this issue, current research focuses on the following directions:

- Introduction of RIS: By programmatically controlling the amplitude and phase of electromagnetic waves, RIS can dynamically optimize signal propagation paths, enabling non-line-of-sight (NLoS) transmission and significantly extending coverage.
- Deployment of Relay Nodes and Distributed Antenna Systems (DAS): Strategically placing relay devices in complex environments can establish multi-hop communication links, compensating for coverage gaps caused by line-of-sight limitations.
- Integration with Low-Frequency Bands: Employing a hybrid communication architecture that combines sub-6 GHz bands for robust connectivity with mmWave bands for high data throughput can achieve a balance between performance and coverage.

### 7.2. Power Consumption and Thermal Management: Heat Dissipation in Highly Integrated Systems

As mmWave antennas evolve towards higher levels of integration—especially in chip-level and package-level integration—RF front-ends, beamforming circuits, and antenna arrays are tightly integrated on the same substrate or within the same package. This results in increased power density and localized hotspots, which threaten device longevity and system stability.

To tackle these thermal challenges, innovative research is being conducted at multiple levels:

- Development of New Thermal Conductive and Dissipative Materials: Materials such as graphene, diamond, and high-conductivity ceramics are being explored to enhance heat transfer efficiency.
- Optimization of Thermal Management Structures: Techniques including microchannel cooling, thermoelectric cooling, and airflow-guiding structures aim to improve overall system thermal performance.
- Low-Power Circuit and Energy-Efficient Algorithm Design: Optimizing RF front-end architectures, reducing beamforming power consumption, and introducing adaptive power management strategies help minimize energy usage.

### 7.3. Cost and Mass Production Challenges: Balancing Performance and Economics

Millimeter-wave antennas often require high-performance, low-loss materials—such as PTFE, hydrocarbon-based materials, LCP, and microwave dielectric ceramics—as well as precision manufacturing processes like LTCC, MEMS, and high-resolution PCB fabrication. These factors not only increase production costs but also complicate large-scale manufacturing. Moreover, varying performance requirements across different application scenarios and the lack of unified design standards hinder widespread commercial deployment.

Key solutions include the following:

- Exploration of Low-Cost Alternative Materials: Modified polymers and flexible printed substrates offer cost-effective alternatives while maintaining acceptable performance levels.
- Promotion of Standardized Manufacturing Processes: Establishing universal packaging specifications and interface protocols for mmWave antenna modules can facilitate industry-wide collaboration.
- Development of Smart Manufacturing and Automated Testing Technologies: Improving production efficiency, reducing manual involvement, and ensuring product consistency and yield.

### 7.4. Standardization and Compatibility Issues: Interoperability Across Vendors

Currently, there is no globally unified technical standard for mmWave communications, especially regarding antenna interfaces, beam management protocols, channel models, and RIS control mechanisms. The lack of interoperability among equipment from different vendors hinders global deployment and ecosystem development.

To promote the maturity and adoption of mmWave communications, it is essential to implement the following:

- Establish Unified International Standards: Led by organizations such as 3GPP, IEEE, and ITU, coordinated efforts are needed to advance standardization across all aspects of mmWave communication systems.
- Enhance Cross-Vendor Collaboration and Interoperability Testing: Joint laboratories and open platforms can validate system compatibility and promote convergence.
- Build Open-Source Toolchains and Simulation Platforms: Supporting modeling, algorithm verification, and system evaluation will lower development barriers and accelerate innovation.

### 7.5. Future Development Directions: Technological Convergence and System Innovation

Looking ahead to the era of 6G and beyond into THz communications, mmWave antennas are expected to evolve towards higher performance, greater intelligence, and more flexibility. Key development trends include the following:

- More Efficient MIMO and Beamforming Algorithms: AI-driven self-learning beam alignment, fast switching, and interference suppression algorithms are becoming mainstream.
- Metamaterials and RIS-Assisted Communications: Metamaterials enable compact broadband antennas, while RIS facilitates dynamic beam control.
- Pre-Research on THz Band Antennas: Early exploration of antenna designs for the 0.1–1 THz band aims to overcome traditional material and process limitations.
- AI-Driven Adaptive Antenna Systems: Deep learning enables real-time environmental sensing and automatic adjustment of antenna parameters to maintain optimal communication states.
- Cost-Effective and High-Stability Manufacturing Processes: Emerging technologies such as flexible electronics, printed electronics, and roll-to-roll manufacturing are being explored for mmWave antenna applications.

## 8. Conclusions

In summary, mmWave antenna technology plays a pivotal role in advancing 5G wireless communication systems by enabling high-speed, low-latency, and large-scale connectivity. This review has provided a comprehensive overview of the state-of-the-art in mmWave antenna design, covering key aspects such as antenna types, MIMO architectures, and advanced beamforming techniques that significantly enhance system capacity and link robustness. Modern integration strategies—such as AiP and chip-level designs—are instrumental in achieving compact, high-performance modules suitable for mass deployment. The selection of appropriate materials—from low-loss substrates like PTFE, hydrocarbon-based materials, LCP, and microwave dielectric ceramics—together with emerging fabrication technologies such as LTCC, 3D printing, and MEMS—further improves both electrical performance and manufacturing scalability. Despite remarkable progress, several technical challenges remain, including signal propagation limitations, thermal management in highly integrated circuits, cost–performance trade-offs in large-scale production, and the need for standardized interoperability across vendors. However, rapid advancements in novel functional materials—such as graphene, metamaterials, and phase-change materials—combined with AI-driven optimization, smart beam management, reconfigurable antenna architectures, and hybrid mmWave–sub-6 GHz systems—are paving the way for next-generation solutions with enhanced adaptability, efficiency, and deployability. Looking ahead, mmWave technology will not only underpin the evolution of 5G and future 6G networks but also expand into diverse application domains, including smart cities, industrial IoT, and space–air–ground integrated communication systems. Therefore, mmWave antennas are poised to become the critical enablers of ultra-high-capacity, low-latency wireless networks, overcoming the spectrum bottleneck and driving the next wave of global digital transformation through ubiquitous, intelligent connectivity that reshapes industries, cities, and human experiences.

## Figures and Tables

**Table 1:** Performance comparison of mmWave microstrip patch antennas.

Ref.	Antenna Type	Substrate Type	Antenna Dimension	Operating Bands (GHz)	Gain (dBi)	Efficiency	Impedance Bandwidth
[14]	Symmetrical E-shaped	Taconic TLY	0.46 mm × 0.70 mm	37.5	8.5	≥85%	45.4%
[15]	Elliptical Slot	Rogers RT5880LZ	16 mm × 16 mm	28	6	94%	N/A
[16]	Square Slot	Rogers RT5880	12 mm × 12 mm	37	8.18	N/A	16.22%
[17]	Multiband Multilayered	Taconic TLY-5	8.9 mm × 10 mm	40–80	8.65	N/A	N/A
[19]	Linear Antenna Array	Roger 5880	17.45 mm × 99.2 mm	24–31	19.88	86%	5.37 GHz

**Table 2:** Performance comparison of waveguide antennas.

Ref.	Antenna Type	Operating Bands (GHz)	Peak Gain (dBi)	Return Loss (dB)	Impedance Bandwidth	Design
[21]	SIW horn	24–28	8.06	−30.89	N/A	Semicircular structure
[22]	SIW H-plane horn leaky wave	22.8 and 26.32	7.3 and 8.1	−33.5	N/A	Dielectric loading and metamaterial structures
[23]	Shared-aperture 2D leaky-wave array	19–34	23.6	N/A	56.6%	A unit cell with two asymmetrical slots
[24]	Slot array	27.5–32.6	28.9	N/A	17%	Ridge gap waveguide and a tapered feeding network
[25]	Full-metal planar array	18.8–30.3	27.7	N/A	46.8%	Double-step double-ridged slot element

**Table 3:** Performance comparison of antenna arrays.

Ref.	Antenna Type	Element Number	Operating Frequency (GHz)	Peak Gain (dBi)	Substrate
[28]	Vivaldi	1 × 4	24.19–29.15, 30.28–40.5	13.2	RT/Duroid 5880
[29]	RIS	256	28.5	19.1	N/A
[30]	Planar	4 × 4	24	20	Rogers 5880 with an MSL feed

**Table 4:** A comparison table of the three processes.

Type	Dielectric Constant	Loss Tangent	Interconnect Density	Cost
LTCC	5–8	0.003	Low	High
eWLB	3.2	0.004–0.035	High	Low
HDI	3–5	0.003–0.01	Medium	Low

**Table 5:** Isolation improvements of different decoupling techniques.

Ref.	Technology	Operating Bands (GHz)	Mutual Coupling Reduction/ Isolation Improvement	Substrate	Relative Design Complexity	Antenna Dimension (mm^2^)
[56]	DGS	3–3.6, 3.6–3.9	Isolation improved to more than 10 dB	FR-4	Low	19.5 mm × 7.4 mm
[57]	PDT	2.45	Mutual coupling reduced to around −40 dB	Rogers RO4350	High	N/A
[58]	Slit structures	3.6	High isolation with more than −25 dB mutual coupling	FR-4	Low	25 mm × 25 mm
[59]	DRAs with vias added vertically	25–27	E-plane and H-plane coupling reduced 19.8 and 22.7 dB, leading to a high isolation level of over 30 dB	Rogers 6010	Low	N/A
[60]	CSRRs	36–50	Isolation improved to 22 dB	Rogers RO4350B	Low	28 mm × 28 mm
[61]	EBG structures	28	−60 dB, −72 dB of peak mutual coupling reduction and −25 dB of isolation improvement	Rogers RO4350B	High	N/A

**Table 6:** Three levels of integration technologies.

Integration Level	Operating Frequency (GHz)	Thickness (mm)	Typical Size	Interconnect Loss	Typical Technologies
Chip-level	<200	0.05–1	μm~mm	Low	CMOS, SiGe
Package-level	<120	0.1–1.5	~mm	Medium	eWLB, FOWLP, HDI
Module-level	<60	0.5–3	mm~cm	High	LTCC, PCB HDI

**Table 7:** Summary of fabrication technologies for mmWave antennas.

Fabrication Technology	Features	Applications
PCB Printing	Mature and cost-effective Suitable for mass production Typical size accuracy: <0.1 mm Frequency range: up to 100 GHz	Mid-to-low-end mmWave devices 5G terminals Consumer electronics
LTCC	Multilayer integration Supports 3D structures Dielectric constant: 3~10 Thermal expansion coefficient: <10 ppm/°C Bending strength: >100 MPa Elastic modulus: >80 GPa Frequency range: up to 300 GHz	RF FEMs mmWave radar 5G base stations Satellite communications
3D Printing	Enables complex geometries Relatively low development cost Supports rapid prototyping Surface roughness: <50 μm Frequency range: up to 300 GHz	Prototype verification Customized small-batch production Novel antenna structures
MEMS	Miniaturized and reconfigurable Enables dynamic control of frequency Actuation voltage: 5–50 V Response time: μs~ms Frequency range: up to 100 GHz	Smart beamforming systems Reconfigurable antennas Wearable devices

**Table 8:** Summary of material selection for mmWave antennas.

Materials		Examples	Advantages
Low-Loss Dielectric Materials	PTFE	RO 3003 (Rogers) RT/Duroid 5880 (Rogers) RF-35 (Taconic)	Stable dielectric properties Good thermal management Compatibility with PCB processes
	Hydrocarbon-based Materials	RO 4350B (Rogers) RO 4350C (Rogers) I-Tera^®^MT40 (Isola)	Stable dielectric properties Low cost and thermal stability Compatibility with PCB processes
	LCP	Vectra^®^ (Celanese) Zenite^®^ (DuPont) Xydar^®^ (SABIC)	Very low dielectric loss Moisture resistance Excellent flexibility for wearable devices
	Microwave Dielectric Ceramics	Al_2_O_3_ Mg_2_SiO_4_ Mg_2_Al_4_Si_5_O_18_	Ultra-low loss tangent High mechanical strength Multilayer integration (LTCC design)
Novel Functional Materials	Graphene	CVD-grown graphene Graphene-based composites	Ultra-high electron mobility Tunable electromagnetic response Supports reconfigurable designs
	Metamaterials	Split-ring resonators Fishnet structures Artificial magnetic conductors	Exotic EM properties (negative refractlon, perfect absorption, etc.) Enhances directivity and bandwidth
	Smart and Phase-Change Materials	Vanadium dioxide (VO_2_) Germanium antimony telluride (GST, Ge_2_Sb_2_Te_5_)	Dynamic tunability via external stimuli Supports programmable RF components and adaptive antenna functions
